# Treatment Patterns and Population Characteristics of Nonpharmacological Management of Chronic Pain in the United States’ Medicare Population: A Scoping Review

**DOI:** 10.1093/geroni/igad085

**Published:** 2023-08-11

**Authors:** Erum Choudry, Kara L Rofé, Kristin Konnyu, Brandon D L Marshall, Theresa I Shireman, Jessica S Merlin, Amal N Trivedi, Catherine Schmidt, Fiona Bhondoekhan, Patience Moyo

**Affiliations:** School of Public Health, Brown University, Providence, Rhode Island, USA; School of Public Health, Brown University, Providence, Rhode Island, USA; Center for Evidence Synthesis in Health, Department of Health Services, Policy and Practice, School of Public Health, Brown University, Providence, Rhode Island, USA; Department of Epidemiology, School of Public Health, Brown University, Providence, Rhode Island, USA; Center for Gerontology and Healthcare Research, Department of Health Services, Policy and Practice, School of Public Health, Brown University, Providence, Rhode Island, USA; Division of General Internal Medicine, University of Pittsburgh School of Medicine, Pittsburgh, Pennsylvania, USA; Division of Infectious Disease, University of Pittsburgh School of Medicine, Pittsburgh, Pennsylvania, USA; Center for Gerontology and Healthcare Research, Department of Health Services, Policy and Practice, School of Public Health, Brown University, Providence, Rhode Island, USA; Department of Medicine, Brown University, Providence, Rhode Island, USA; Department of Physical Therapy, Massachusetts General Hospital Institute of Health Professions, School of Health and Rehabilitation Sciences, Boston, Massachusetts, USA; Department of Epidemiology, School of Public Health, Brown University, Providence, Rhode Island, USA; Center for Gerontology and Healthcare Research, Department of Health Services, Policy and Practice, School of Public Health, Brown University, Providence, Rhode Island, USA

**Keywords:** Evidence synthesis, Medicare, Nonpharmacological pain management, Older adult, Scoping review

## Abstract

**Background and Objectives:**

Clinical practice guidelines recommend noninvasive nonpharmacological pain therapies; however, reviews that assess the literature pertaining to nonpharmacological pain management among older adults and people with long-term disabilities who are disproportionately affected by pain are lacking. This scoping review aimed to systematically map and characterize the existing studies about the receipt of noninvasive, nonpharmacological pain therapies by Medicare beneficiaries.

**Research Design and Methods:**

We conducted a literature search in MEDLINE (PubMed), CINAHL (EBSCO), SocINDEX (EBSCO), Cochrane Library, Web of Science citation indices, and various sources of gray literature. The initial search was conducted on November 2, 2021, and updated on March 9, 2022. Two independent reviewers screened titles, abstracts, and full texts for inclusion and extracted the characteristics of the studies, studied populations, and nonpharmacological pain therapies. Data were summarized using tabular and narrative formats.

**Results:**

The final review included 33 studies. Of these, 24 were quantitative, 7 were qualitative, and 2 were mixed-methods studies. Of 32 studies that focused on Medicare beneficiaries, 10 did not specify the Medicare type, and all but one of the remaining studies were restricted to fee-for-service enrollees. Back and neck pain and arthritis were the most commonly studied pain types. Chiropractic care (*n* = 19) and physical therapy (*n* = 17) appeared frequently among included studies. The frequency and/or duration of nonpharmacological treatment were mentioned in 13 studies. Trends in the utilization of nonpharmacological pain therapies were assessed in 6 studies but none of these studies went beyond 2008.

**Discussion and Implications:**

This scoping review found that manipulative therapies, mainly chiropractic, have been the most widely studied approaches for nonpharmacological pain management in the Medicare population. The review also identified the need for future research that updates trend data and addresses contemporary issues such as rising Medicare Advantage enrollment and promulgation of practice guidelines for pain management.

**Translational Significance:** Literature reviews that address the utilization of nonpharmacological pain treatments within the Medicare population are lacking. This gap is particularly concerning because nonpharmacological treatments are widely recommended and that Medicare enrollment, comprising mainly older adults, is increasing. The main outcome of this study was to summarize the literature on receipt of nonpharmacological pain therapies among Medicare enrollees. There is a need for empirical studies that utilize contemporary data given rising Medicare Advantage enrollment and evolving pain treatment guidelines. Our findings contribute to systematic reviews and other research to inform practice and policy around nonpharmacological approaches to pain management in Medicare.

Chronic pain, defined as pain lasting at least 3 months, affects approximately one-third (31%) of older adults in the U.S., and this prevalence increases with advancing age (1). The prevalence of chronic pain has also been reported to be particularly high (52.4%) in adults with a disability (2). Chronic pain is one of the most common reasons for seeking medical care, particularly in later life. With the rising proportion of the American population aged 65 years or older, there will be a progressive increase in Medicare enrollment (3). Previous studies have reported that Medicare beneficiaries have a higher prevalence of chronic pain compared to the general population, attributed to factors like advanced age and disabilities, highlighting the disproportionate impact of chronic pain on Medicare beneficiaries (4,5).

Significant shifts are expected in Medicare, with enrollment in this federal health insurance program for older adults and individuals with long-term disabilities projected to nearly double between 2016 and 2060 (6,7). Additionally, there are rapid increases in the share of Medicare beneficiaries who are enrolled in a private plan option (Medicare Advantage) for Medicare health coverage rather than a fee-for-service option (Traditional Medicare). An estimated 29 million individuals were enrolled in Medicare Advantage in April 2022, representing about 46% of all beneficiaries in Medicare (8). The range of nonpharmacological pain therapies covered and the extent of such insurance coverage, differ between Traditional Medicare and Medicare Advantage with the latter considered to offer more comprehensive and generous coverage (7). Hence, the U.S. and Medicare may be faced with growing demands for pain management among its aging population and considerations should be given toward the delivery of comprehensive pain management in both Traditional Medicare and Medicare Advantage. Simultaneously, in response to the opioid epidemic, clinical practice guidelines prioritize nonpharmacological therapies over opioid medications to manage chronic pain (9,10). Therefore, understanding the characteristics of Medicare beneficiaries with chronic pain and the attributes of their use of nonpharmacological approaches to pain is a critical step toward the delivery of equitable and guideline-recommended care for chronic pain.

Opioids emerged as one of the most prescribed pharmacologic agents for chronic noncancer pain management in the U.S. since the early 1990s (11,12). However, the safety concerns and questionable effectiveness of opioid therapy for pain, especially when used long-term, have led to the promulgation of several guidelines recommending more judicious and safer use of opioids in order to minimize opioid-related risks, including from the American College of Physicians (ACP) (13), and Veterans Health Administration (VHA) (14). In November 2022, the Center for Disease Control and Prevention (CDC) issued its updated clinical practice guideline for prescribing opioids for pain, which advocates limiting opioid prescriptions and promotes the use of nonpharmacological approaches as first-line therapies for treating chronic pain (10). Research on the effectiveness of noninvasive and nonpharmacological pain therapies indicates wide variability in the size of the effect and strength of evidence on the effectiveness of these strategies on pain control and functional ability (15–17). However, evidence from systematic reviews suggests that nonpharmacological therapies (including spinal manipulative, massage, acupuncture, and multidisciplinary rehabilitation) confer small-to-moderate benefits, though usually short-term, and mainly for chronic low back pain (16). Although questions remain regarding which patient subgroups benefit most from a specific type of nonpharmacological treatment, access and use of nonpharmacological, nonopioid alternatives are recommended for the comprehensive management of chronic pain. Therefore, it is important to characterize the existing patterns of nonpharmacological use to help identify opportunities to advance policy, practice, and research. The historic influence of Medicare in shaping health care policy and practice in the U.S. suggests that Medicare’s coverage decisions have far-reaching implications, affecting not only Medicare beneficiaries but also individuals with private insurance (18,19). This serves as further rationale for the current study.

To our knowledge, no comprehensive review has examined the utilization characteristics of nonpharmacological therapies for chronic pain specifically within the Medicare population. Although there are several existing reviews on the effectiveness of nonpharmacological therapies in older adults (15,16,20,21), there remains a lack of literature reviews that specifically address the utilization of these treatments within the Medicare population. To this end, this scoping review systematically scanned and characterized existing studies that examined the type, duration, frequency, and setting of nonpharmacological pain therapies among older adults and people with disabilities who are eligible for or enrolled in Medicare. We also evaluated the use of nonpharmacological pain treatments as alternatives or complements to opioid therapy and additionally assessed the outcomes examined in these studies and the demographic and clinical profiles of the studied populations.

## Method

This scoping review was conducted in accordance with the Joanna Briggs Institute (JBI) methodology (22) and followed the extension of the Preferred Reporting Items for Systematic Reviews and Meta-Analysis framework for scoping reviews (PRISMA-SCR) (23). For the purposes of this review, we published (24) and registered our protocol in Open Science Framework (https://osf.io/h7bwc/). The detailed search strategy for the review can be found in the study protocol (24).

### Research Questions

The following research questions guided this scoping review:

I. What are the characteristics (type, duration, frequency, setting) of nonpharmacological pain treatment services that are utilized in the Medicare population?
II. Which population subgroups are addressed and what outcomes were evaluated?
III. Are nonpharmacological pain treatment modalities used as alternatives or complements to medications?

### Concept

The concepts that were assessed for the scoping review are utilization of nonpharmacological treatments for chronic noncancer pain. Given that not all studies may report the chronicity of pain, we allowed for studies that focused on pain types that are often chronic (e.g., musculoskeletal conditions such as back pain) to be included. The selection of nonpharmacological treatments included in the review was informed by guidelines promoted by several health care agencies and professional societies, including the ACP, CDC, Agency for Healthcare Research and Quality (AHRQ), the Academic Consortium for Integrative Medicine and Health, the American Academy of Family Physicians, Veterans Health Association, the Department of Health, and Human Services, The American Society of Anesthesiologists, and the Office of the Army Surgeon General. Specifically, this scoping review focused on 14 nonpharmacological pain treatments recommended by 3 or more of these organizations (24). We selected this criterion due to its alignment with the guidelines recommended for high levels of evidence, as reported by Ackley et al. (25). The 14 selected nonpharmacological approaches were exercise, massage, Tai chi, yoga, mindfulness-based stress reduction (or other mindfulness techniques), multidisciplinary rehabilitation, progressive relaxation (or other relaxation techniques), acupuncture, low-level laser therapy, biofeedback, psychological therapies, manipulative therapy, electrical stimulation (ie, TENS: transcutaneous electrical nerve stimulation), and myofascial release.

### Inclusion and Exclusion Criteria

This scoping review evaluated U.S.-based studies of older adults (≥65 years) with unspecified insurance providers and Medicare-enrolled older adults and individuals with disabilities who experience chronic, noncancer pain, and receive noninvasive nonpharmacological pain management. The review included studies of Medicare fee-for-service and Medicare Advantage beneficiaries who were entitled for benefits under Medicare based on their age or presence of a qualifying disability. Because the Medicare program primarily provides health insurance to people older than 65 years, publications that focused on older adults living in the U.S. without indicating their enrollment status in the Medicare program were also included.

Medicare beneficiaries who were adults with disabilities were also included in the review. However, articles that did not identify Medicare enrollment for disabled individuals under the age of 65 were excluded from the study.

### Study Designs

This scoping review focused on empirical qualitative and quantitative studies in real-world settings rather than experimental conditions. A variety of study designs were included in the search, such as analytical observational studies including prospective and retrospective cohort studies, case-control studies, cross-sectional studies, and quasi-experimental studies. Single-site case series, individual case reports, Medicare demonstration projects, randomized and nonrandomized controlled trials, and studies that analyzed national survey data or large health care databases but did not have subgroup analyses of older adults or Medicare beneficiaries were excluded. Newsletters, expert opinions, and perspective papers were also excluded because they were considered unlikely to contain the information required for data extraction and to contribute little generalizable knowledge.

### Search Strategy

A comprehensive electronic search was carried out to identify published and unpublished studies in English language from January 1990 to March 2022 on MEDLINE (PubMed), CINAHL (EBSCO), SocINDEX (EBSCO), Cochrane Library, and the following citation indices from the Web of Science Core Collection: Science Citation Index Expanded, Social Sciences Citation Index, Arts and Humanities Citation Index, and Emerging Sources Citation Index. A separate search of gray literature was also performed in ProQuest Dissertations and Theses Global, the OECD iLibrary, and through the Centers for Medicare and Medicaid Services (CMS) website. Of the types of gray literature that may have been obtained, dissertations and white papers were considered for inclusion, whereas abstracts were not due to their limited information. The initial electronic database searches took place on November 2, 2021, and an updated search was completed on March 9, 2022.

### Selecting the Relevant Literature

Following the electronic search, we imported all identified articles into Zotero 5.0.96.3 (Corporation for Digital Scholarship and Roy Rosenzweig Center for History and New Media, VA, USA) and removed duplicates. After retrieving the full texts of potentially relevant studies, we used Abstrackr (Association for Computing Machinery, NY, USA) to import the citation details. Titles and abstracts were screened by 2 independent reviewers (K.R. and P.M.) against eligibility criteria for the review. Full texts of potentially relevant records were retrieved and screened by 2 independent reviewers. All conflicts were resolved by a consensus discussion.

### Data Extraction Process

A comprehensive data extraction spreadsheet was developed by the authors following guidelines provided by the JBI methodology for scoping reviews format. Microsoft Excel was used for data charting and storage. Studies with multiple records were linked (i.e., studies reported the same sample size and population in more than one study or published by same authors in same year) by describing the studies as *a* and *b*. Two reviewers extracted data independently; all conflicts were resolved through discussion and consensus.

### Data Items Extracted

The data items extracted into spreadsheets included but were not limited to the following parameters: (i) study identification, for example, study author(s), publication year, and complete citation; (ii) study characteristic, for example, study aims, design, clinical care setting, geographic location, and years of data acquisition; (iii) sample characteristics: for example, age, sex, race, and ethnicity; (iv) type of treatment modalities and their characteristics; and (v) outcomes assessed and findings.

### Result Reporting Process

After extracting data from individual studies, we developed categories to facilitate the summarization of elements that related to describing the studies and research questions. We used frequencies and percentages to describe the characteristics of the included studies and the characteristics of individuals from the included studies. Additionally, we report pharmacologic and nonpharmacological treatment modalities, their characteristics, and outcomes assessed by the included studies. The results are presented in narrative, diagrammatic, and tabular formats.

A formal quality assessment of the included studies was not conducted given the descriptive nature of the objectives and that the PRISMA-SCR checklist does not require a quality appraisal for a scoping review. The purpose of the scoping review was to provide a comprehensive summary of the evidence, serving as a potential preliminary step to future systematic reviews where a quality assessment would be essential.

## Results

### Characteristics of Included Studies

**Figure 1.**
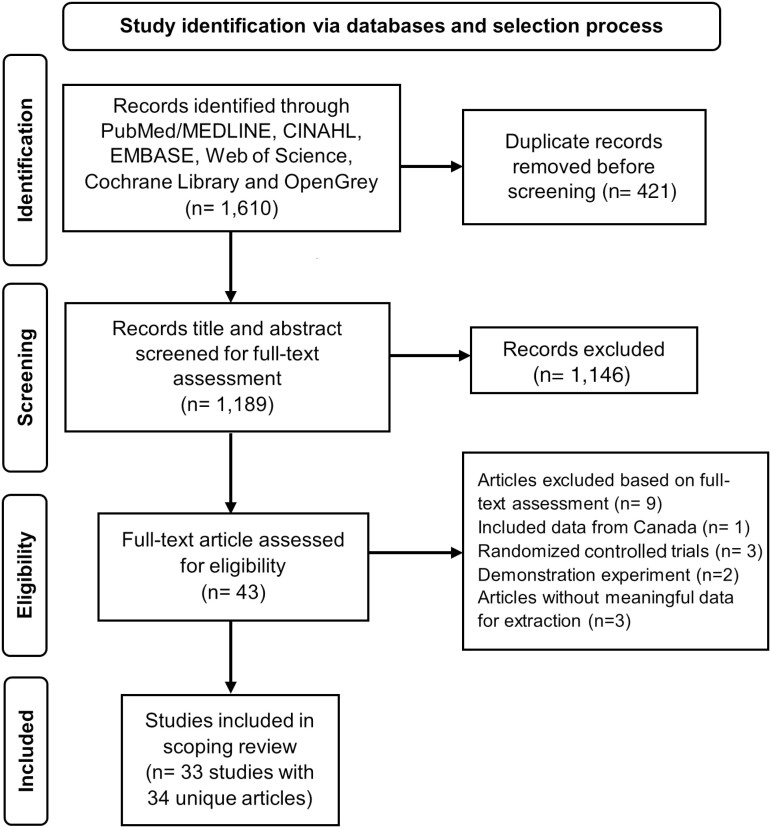
PRISMA flow diagram of the identification and selection of studies for the scoping review.

The literature searches yielded 1 189 potentially relevant articles after removing the duplicates. These articles were subsequently screened by title and abstract in accordance with the inclusion criteria, removing 1 146 studies. A total of 43 articles were selected for full-text assessment; of these, 9 were excluded based on exclusion criteria. See the PRISMA flow diagram (Figure 1) for information on each stage of the identification and selection process. During the full-text analysis phase, 3 randomized controlled trials (RCTs) were excluded from the study because they are often highly controlled and selective and were deemed not to reflect the real-world environments, populations, or health care use patterns. Two Medicare demonstration projects were also excluded as they mimic experimental designs, which do not reflect real-world contexts of clinical care and policy. We deemed demonstration projects to belong in a different category of studies than those based on nonexperimental conditions, and thus warrant consideration in a separate review tailored to identify and extract information from implementation studies and RCTs. In addition, 3 more articles were excluded because they included data and findings, which were not defined as an inclusion criterion in the protocol of this scoping review. Finally, 1 more article was excluded as it included data from Canada, which was beyond the scope of this review. There were 33 research studies, representing 34 distinct articles, included in this scoping review.

Table 1 summarizes the characteristics of included studies, of which 32 were journal articles and 1 was a dissertation. The median year of data acquisition was 2005 with a range between 1990 and 2018, and the publication years ranged from 1992 to 2022. The scoping review included 11 cross-sectional studies and 22 cohort studies. Furthermore, this scoping review identified 73% quantitative, 21% qualitative, and 6% mixed method studies. Out of 33 studies, only 1 study specifically included the Medicare Advantage enrollees, 22 studies excluded Medicare Advantage, and 10 studies did not specify. More than three-quarters (79%) of studies included data from a national level, 6% from multiple states, and 15% from a single state. Only 4 (12%) studies included disabled adults with the age range between 65 and >85 years. Approximately 55% of studies specified the mid or lower back region as the source of pain followed by head or neck region (18%).

**Table 1.** Summary Characteristics of Included Studies in the Scoping Review

Characteristic	*n* (%)
Total number of studies	33
Publication type	
Journal article	33 (97)
Dissertation	1 (3)
Publication years	
Range	1992, 2022
Years of data acquisition	
Median	2005
Range	1990, 2018
Study design	
Cross-sectional study	11 (33)
Cohort study	22 (67)
Study methods	
Quantitative	24 (73)
Qualitative	7 (21)
Mixed methods	2 (6)
Medicare advantage	
Yes	1 (3)
No	22 (67)
Not specified	10 (30)
Disabled adults	
Yes	4 (12)
No	29 (88)
Clinical care setting^*^	
Outpatient	31 (94)
Inpatient	3 (9)
Home	6 (18)
Long-term care	2 (6)
Others (not specified)	1 (3)
Geographic location	
Single state level	5 (15)
Multistate level	2 (6)
National level	26 (79)
Location of pain^*^	
Mid or lower back region	18 (55)
Head or neck region	6 (18)
Upper or lower extremities	5 (15)
Others	2 (6)
Not reported	11 (33)

*Note*:

^*^Some studies included multiple clinical care settings and multiple locations of pain.

### Population Characteristics of Included Studies

Twenty-nine studies reported overall sample sizes that ranged from 551 to 17.7 million (26–55). Twenty studies reported a mean age between 69.5 and 81.7 years (27,28,30–34,36,38–44,46,48–50,56,57). Three studies reported the age range for participants included in their studies (45,57,58). Twenty-nine studies provided the biological sex of the study population and the percentage of females ranged between 55% and 73% in the included studies (26–50,52,54,56–59). Six of the included studies did not provide any race and ethnicity characteristics of participants (34,42,51,53,55,56). Lieberz et al. (39) and Albert et al. (27,28) reported the highest number of White (98%) and Black (52%) populations, respectively, as shown in Table 2.

**Table 2.** Population Characteristics of Studies Included in the Scoping Review

First Author, Year	Sample Size (*n*)	Age, Mean (*SD*) or Range	Female (%)	Race and Ethnicity (%)^¶^					
				White	Black	Hispanic	AAPI	AI/AN	Others
Albert, 2008	551	73.4(NR)	61	48	52	NR	NR	NR	NR
Astin, 2000	1 597	NR	55	76	3	6	11	NR	4
Davis, 2015	17.7 M	76.5(NR)	59	88	7	NR	NR	NR	5
Davis, 2019	84 679	77(±7.2)	68	NR	4	NR	NR	NR	96
Davis, 2021	39 278	75.5(NR)	61	97	NR	NR	NR	NR	2
De Heer, 2016	413 608	70.6(±13.4)	65	84	10	NR	NR	NR	6
Fritz, 2011	1 840	74.2(±6.3)	65	NR	NR	NR	NR	NR	NR
Gell, 2017	7 487	NR	58	68	21	6	NR	NR	3
Hufstader, 2009	181	75.6(±7.2)	60	50	NR	NR	NR	NR	48
Jordan, 2000	937	NR	56	93	7	NR	NR	NR	NR
Karmali, 2020	65 101	75.6(±7.7)	66	85	7	NR	NR	NR	8
Latham, 2008	*n*/year^*^	NR	67	85	NR	NR	NR	NR	15
Lieberz, 2020	706	69.5(±10.9)	71	98	NR	NR	NR	NR	2
Ly, 2020	162 238	76.6(NR)	71	78	7	8	6	NR	1
Mayer-Oakes, 1992	809	73.5(NR)	56	88	NR	NR	NR	NR	12
Musich, 2020	3 994	77.2(NR)	68	NR	NR	NR	NR	NR	NR
Ngo, 2009	12 983^†^	74.9(NR)	68	82	9	6	NR	NR	2
Sclafani, 2017	95 647	72.8(±9.8)	69	88	8	2	1	0.4	1
Standaert, 2020	170 011	70.7, 70.2	60	88	7	NR	NR	NR	5
Stevans, 2017	52 504	76.6(NR)	73	79	8	8	4	0.4	1
Thorpe, 2021	5 613 384	NR	61	86	8	2	NR	NR	4
Weeks, 2016	40 720	75.4(NR)	62	89	NR	NR	11.4	NR	NR
Weigel, 2010	5 510	77.4(NR)	62	85	10	4	NR	NR	1
Weigel, 2012	7 447	NR	NR	NR	NR	NR	NR	NR	NR
Weigel, 2013	5 871	75.8(NR)	62	95	2	3	NR	NR	NR
Weigel, 2014a	12 170	NR	63	91	5	2	NR	NR	2
Weigel, 2014b	1 057	81.7(NR)	67	89	7	4	NR	NR	NR
Weiner, 2006	*n*/year^‡^	74.8(NR)	59	NR	NR	NR	NR	NR	NR
Whedon, 2012	*n*/year^§^	73.8, 73.9	59	96–97	1–2	<1	<1	<1	<1
Whedon, 2013	5.0 to 5.4 M	NR	NR	NR	NR	NR	NR	NR	NR
Whedon, 2021a	*n*/year^∥^	NR	NR	NR	NR	NR	NR	NR	NR
Whedon, 2021b	28 160	72.6, 73.1	73	90	NR	NR	NR	NR	13
Whedon, 2022	55 949	NR	68	76	9	NR	NR	NR	14

*Notes*: AAPI = Asian American Pacific Islander; AI/AN = American Indian or Alaska Native; F = females; M = million; NR = not reported.

^*^1995 = 7 978; 1999 = 7 863; 2001 = 7 973.

^†^Patients with arthritis.

^‡^Study 1: (1991 = 16 067 386; 1992 = 17 088 116; 1993 = 18 021 334; 1994 = 18 865 557; 1995 = 19 652 799; 1996 = 20 099 417; 1997 = 20 326 859; 1998 = 20 435 026; 1999 = 20 572 393; 2000 = 21 039 207; 2001 = 22 153 102; 2002 = 22 892 876). Study 2: (2000 = 32 616; 2001 = 35 946; 2002 = 34 408).

^§^2002 = 1 630 950; 2003 = 1 689 035; 2004 = 1 767 655; 2005 = 1 821 430; 2006 = 1 803 055; 2007 = 1 759 615; 2008 = 1 721 160.

^∥^2007 = 45 475 990; 2008 = 46 607 357; 2009 = 47 554 332; 2010 = 48 730 667; 2011 = 50 330 565; 2012 = 52 163 927; 2013 = 53 800 907; 2014 = 55 597 355; 2015 = 57 063 713.

^¶^The cells marked as “NR” in race and ethnicity category indicates that the study did not report data or did not specify information on that particular category. Percentages may add up to 100% based on the population included in the study. However, studies may not report on all racial and ethnicity categories, so it is possible that some subgroups are not represented in the reported percentages.

### Treatment Modalities Included in Studies

Supplementary Table 1 outlines the specific pain treatment modalities addressed in each study included in the review. In addition to the 14 nonpharmacological therapies of interest, we report on pharmacologic, surgical, and other nonpharmacological strategies identified in the review. Twelve studies reported on opioid medication use (28,32,36–40,42,46,48,54,57), and 6 studies reported on nonopioid medication use, which includes the utilization of nonsteroidal antiinflammatory drugs (NSAIDS), acetaminophen, etc. (28,36,37,40,46,48). Ten studies reported on the use of muscle relaxants, antidepressants, and spinal and knee injections as pharmacologic treatment modalities (28,37,39,40,42,44–46,48,56). Thirteen studies in our review reported data on usage of both pharmacologic and nonpharmacological treatment modalities (28,29,32,36–40,42,46,48,54,56,57). Among them, Albert et al. (27) reported that nonpharmacological self-management modalities do not substitute for medication usage. Another study observed an increase in the rate of visits to primary care physicians due to reduced access to chiropractic care (32). Stevans et al. (46) and Whedon et al. (54) reported that use of rehabilitation services is associated with significantly lower use of pharmacologic treatment modalities. Opioid medication use was considered either descriptively, as a covariate, or as an outcome measure in 12 studies (28,32,36–40,42,46,48,54,57).

Among noninvasive, nonpharmacological approaches identified from pain treatment guidelines and recommendations, 33 studies reported data on manipulative therapy, which included physical therapy (PT), chiropractic care, or spinal manipulative therapy (26,28–59). Five studies reported data on exercise (27,28,37,42,46), 4 studies included data on massage (28,29,36,37), and only 1 study reported biofeedback (37). None of the studies reported data on Tai chi, yoga, low-level laser therapy, multidisciplinary rehabilitation, electrical stimulation, and myofascial release.

Regarding additional noninvasive, nonpharmacological treatment modalities, 6 studies reported data on diet and nutrition/supplements (27–29,36,37,46). Further, 6 studies reported data on occupational therapy (35,38,41,43,47,59), 4 studies reported data on home/herbal remedies (27–29,36), and 3 studies reported data on ice and heat therapy (27,28,37). Single separate studies presented data on Chinese medication, chelation therapy, homeopathy, ayurvedic medicine, naturopathy, therapeutic ultrasound (29), speech therapy (35), and self-care strategies (27). Six studies reported data on surgical treatment modalities in combination with pharmacologic or nonpharmacological treatment modalities (32,44–46,48,56).

### Characteristics of Noninvasive Nonpharmacological Treatment Modalities

Table 3 presents the intensity (frequency, duration, and prevalence) of noninvasive nonpharmacological treatment modalities reported by the included studies. Eight studies provided data on frequency by reporting mean visits (29,34,39,47–51). Furthermore, 10 studies provided data on the duration of the noninvasive nonpharmacological treatment modalities (33–35,39,43,44,49–51,59). Twenty-one studies did not provide any data on frequency and duration of the noninvasive nonpharmacological treatment modalities (26–28,30–32,36–38,40–42,45,46,52–58) and only 5 studies reported both (34,39,49–51). Twenty-three studies reported data on prevalence of the noninvasive nonpharmacological treatment modalities (26–29,33,35–38,40,42–44,47,49,50,52–54,56–59).

**Table 3.** Characteristics of Noninvasive Nonpharmacological Treatment Modalities Reported by Included Studies

First Author, Year	Frequency	Duration	Prevalence
Albert, 2008a	NR	NR	Prevalence of self-care behaviors
Albert, 2008b	NR	NR	Prevalence of self-care behaviors
Astin, 2000	Mean of CAM visits	NR	Percentages of use of specific CAM therapies^*^
Davis, 2015	NR	NR	NR
Davis, 2019	NR	NR	NR
Davis, 2021	NR	NR	NR
De Heer, 2016	NR	Percentages of duration for hospital admission (PT vs non-PT)	Percentages of beneficiaries with LBP utilized PT
Fritz, 2011	Mean of PT visits	Median of time between initial and final PT visit	NR
Gell, 2017	NR	Duration in months for rehabilitation (PT, OT, and ST)	Percentages of older adults received rehabilitation services in the outpatient setting, and at home
Hufstader, 2009	NR	NR	Percentages of preferred treatment modalities^†^
Jordan, 2000	NR	NR	Percentages of use of complementary, and self-care strategies^‡^
Karmali, 2020	NR	NR	Percentages of use of treatment modalities^§^
Latham, 2008	NR	PT and OT units (each unit has duration of 15 min)	Percentage of individuals receiving PT or OT by setting
Lieberz, 2020	Mean of PT visits	Mean duration of the PT episode of care	NR
Ly, 2020	NR	NR	Percentages of patients with LBP received PT
Mayer-Oakes, 1992	NR	NR	NR
Musich, 2020	NR	NR	Percentages of patients use PT stratified by income
Ngo, 2009	NR	PT and OT units (each unit has duration of 15 min)	Percentages of patients receiving PT
Sclafani, 2017	NR	PT units (each unit has duration of 15 min)	Percentages of patients receiving PT
Standaert, 2020	NR	NR	NR
Stevans, 2017	NR	NR	NR
Thorpe, 2021	Mean PT and OT visits with and without CP	NR	Percentages of patients receiving PT and OT
Weeks, 2016	Mean of CMT visits	NR	NR
Weigel, 2010	Mean of CMT visits	Percentages of patients with chiropractic visits during specific duration	Mean annual and period prevalence of chiropractic use
Weigel, 2012	Mean of CMT visits	Mean chiropractic care episode duration	NR
Weigel, 2013	NR	NR	Percentages of patients receiving chiropractic care
Weigel, 2014a	NR	NR	Average annual prevalence of Medicare beneficiaries using chiropractic
Weigel, 2014b	Mean of CMT visits	Mean days in chiropractic care episodes	Percentages of patients receiving chiropractic care
Weiner, 2006	NR	NR	Prevalence of patients received PT
Whedon, 2012	NR	NR	Percentages of patients using chiropractic care stratified by demographic characteristics
Whedon, 2013	NR	NR	Estimated total number of chiropractic users
Whedon, 2021a	NR	NR	NR
Whedon, 2021b	NR	NR	Percentages of patients received SMT
Whedon, 2022	NR	NR	Number of patients received chiropractic care

*Notes:* CAM = complementary/alternative medicine; CMT = chiropractic manipulative treatment; CP = cerebral Palsy; LBP = lower back pain; NR = not reported; OT = occupational therapy; PCP = primary care physician; PT = physiotherapy; SMT = spinal manipulative therapy; ST = speech therapy.

^*^CAM therapies include herbal medicine, chiropractic, massage, acupuncture, naturopathy, Chinese medicine, guided imagery, homeopathy, ayurvedic medicine, and chelation therapy.

^†^Preferred treatment modalities = massage, acupuncture, chiropractic, prayer/spiritual healing, herbal/mineral.

^‡^Complementary, and self-care strategies = chiropractor, exercise, massage, relaxation, biofeedback, mindfulness-based stress reduction, prayers, jewelry, ice and heat therapy, and diet.

^§^Treatment modalities = PT, OT, and psychological therapy.

### Outcomes Assessed by Included Studies

The most common outcomes assessed by the included studies were nonpharmacological treatment use (*n* = 17 studies) (29,30,32–37,40,41,43,44,47,49,51,56,59), followed by health and functional status, which was assessed by 12 studies as shown in Table 4 (26,27,29,35,37,41,42,46,47,50,52). Other outcomes that were evaluated included cost and service utilization (*n* = 9) (30–32,42,44,45,48,53,56), medication use (*n* = 8) (28,36–38,40,46,54,57), pain (*n* = 5) (27,29,34,42,54), provider supply or availability (*n* = 3) (38,53,55), and social isolation and connectivity (*n* = 3) (27,41,42).

**Table 4.** Outcomes Assessed by Included Studies

First Author, Year	Outcome assessed										
	Pain	Self-Management Behaviors	Health and Functional Status	Mental Health	Social Isolation and Connectivity	Nonpharmacological Treatment Modalities	Pharmacologic Treatment Modalities	Cost and Services Utilization	Provider Supply or Availability	Time Trends in Nonpharmacological Treatment Use	Others
Albert, 2008a	X	X	X	X	X						X
Albert, 2008b		X					X				
Astin, 2000	X		X			X					X
Davis, 2015						X		X			
Davis, 2019								X			
Davis, 2021						X		X			
De Heer, 2016						X					
Fritz, 2011	X					X					
Gell, 2017			X	X		X					X
Hufstader, 2009						X	X				X
Jordan, 2000		X	X			X	X				
Karmali, 2020							X		X		
Latham, 2008						X				X	
Lieberz, 2020			X								
Ly, 2020						X	X				
Mayer-Oakes, 1992		X	X	X	X	X					
Musich, 2020	X		X	X	X			X			X
Ngo, 2009						X				X	
Sclafani, 2017						X		X		X	X
Standaert, 2020								X			
Stevans, 2017			X				X				
Thorpe, 2021			X			X					
Weeks, 2016								X			
Weigel, 2010						X				X	
Weigel, 2012						X					
Weigel, 2013			X	X							
Weigel, 2014a			X								X
Weigel, 2014b			X	X							
Weiner, 2006						X		X			
Whedon, 2012										X	
Whedon, 2013								X	X	X	
Whedon, 2021a									X		
Whedon, 2021b							X				
Whedon, 2022	X						X				
Total	5	4	12	6	3	17	8	9	3	6	7

*Note*: *X* = outcome assessed by the included studies (*the color is added for visual display and does not provide unique information*). Others = health engagement control strategies, prevalence and effect of underlying disease, resilience, patient-reported rehabilitation goal attainment, satisfaction with patient care, and modalities for which members wanted third-party coverage.

Time trends in nonpharmacological treatment use were assessed by 6 including studies (43,44,49,53,58,59). Five studies reported generally small increases in the overall trend in the use of nonpharmacological treatment modalities, specifically for chiropractic care and PT (43,49,53,58,59). Latham et al. reported that the percentage of Medicare beneficiaries who received either PT or occupational therapy increased from 15.6% in 1995 to 19.6% in 2001 (59). Weigel et al. reported that the proportion of the high-volume chiropractic user group (users that exceeded the “soft cap” of 12 visits in a calendar year) grew from 4.6% in 1993 to 15% by 2007 (49). In a study by Whedon et al., the total number of chiropractic users increased from 1.6 million to 1.7 million between the years 2002 and 2008 (58). However, the most recent year of data for which temporal trends were evaluated was 2008. One study by Sclafani et al. (44) used data from 2000 through 2011 focusing on the utilization of nonpharmacological treatment modalities with each subsequent year after the first year of diagnosis of underlying disease and found that the utilization decreased with each year. Other studies that evaluated trends considered changes in the racial diversity of providers, provider supply, and costs of nonpharmacological treatments to Medicare primarily focusing on chiropractic care providers. In the most contemporary study evaluating trends, Whedon et al. (55) used Medicare data from 2007 to 2015 and reported wide geographic variation in the supply of clinicians who bill for spinal manipulation services to Medicare ranging from 20 per 100 000 beneficiaries in the District of Columbia to 260 per 100 000 beneficiaries in North Dakota. The study additionally found that the overall supply of spinal manipulation providers under Medicare is declining, whereas the supply of nonchiropractors who provide spinal manipulation services is growing.

## Discussion

In this scoping review, we aimed to identify and synthesize the literature on the use of nonpharmacological treatment modalities for chronic pain management among Medicare-enrolled and Medicare-eligible adults in the U.S. The review deliberately sought evidence from outside experimental contexts such as RCTs and CMS demonstration projects, in order to gain an understanding of nonpharmacological pain care in real-world contexts. Several findings that respond to our research questions stood out from the 33 empirical studies included in this scoping review (26,28–59). First, all studies addressed aspects of manipulative therapy such as PT, chiropractic care, and/or spinal manipulative therapy, which were delivered in outpatient settings. The frequency and duration of these services was described in one-third of these studies. Second, Medicare Advantage enrollees were often excluded from these studies and were explicitly included in only one study included in the review. Demographically, it was notable that information about race and ethnicity was often not reported for minority populations, particularly Asian American or Pacific Islander persons and American Indian or Alaska Native persons. Third and finally, there is a lack of studies that evaluate more recent trends in the utilization of nonpharmacological pain treatment, particularly amid the national landscape of declining opioid prescriptions and new clinical practice guidelines over the past decade.

Eighteen (55%) of the studies focused on individuals with back pain, making back pain the most common location of pain observed and reported on, which aligns with the fact that back pain is a top reason for physician visits in the U.S. (60). This finding is also potentially explained by Medicare coverage for some nonpharmacological pain treatment such as chiropractic and acupuncture services, being restricted to individuals with chronic low back pain. Therefore, studies that rely on Medicare reimbursement data would be skewed toward observing claims involving back pain.

In this scoping review, only 2 studies provided data on acupuncture (Astin et al. (29) and Hufstader et al. (36)) and both studies were published more than a decade prior to 2020 when CMS began providing Medicare coverage for acupuncture (61). It is worth noting that, in addition to Medicare coverage for acupuncture currently being limited to the management of chronic low back pain, coverage is capped at 12 acupuncture visits within 90 days, with an additional 8 visits allowed contingent on showing clinical improvement (62). New coverage for acupuncture in Medicare policy is an important step toward broadening access to nonpharmacological treatment modalities, which are widely recommended by clinical practice guidelines; however, it is essential to address barriers to the dissemination and implementation of acupuncture, which might hamper the full realization of the benefits of new coverage for acupuncture. According to Liou et al., there are approximately 38 000 licensed acupuncturists in the U.S.; however, nearly half of them practice in New York City, Florida, and California, whereas other states such as Ohio, Kentucky, and Michigan have fewer than 2 acupuncturists per 100 000 people (63). In addition to considerations of geographic availability of acupuncturists, most Medicare providers are unfamiliar with acupuncture and might not be fully equipped to deliver acupuncturist supervision necessary for Medicare coverage. Gaps in provider knowledge and self-efficacy also restrict providers from effectively educating and counseling patients on the benefits of acupuncture in pain management (63). These barriers to dissemination and implementation may also extend to the delivery of PT and chiropractic services covered by Medicare and deserve attention.

This scoping review found a lack of studies on the use of certain noninvasive, nonpharmacological treatment modalities that are widely recommended including yoga, Tai chi, laser therapy, multidisciplinary rehabilitation, electrical stimulation, and myofascial release for chronic pain management in the Medicare population. As there are research studies and clinical practice guidelines that suggest these interventions have utility in the management of chronic pain, it is possible that enhanced access to, and increased acceptance of, these treatments as part of a comprehensive approach to pain management may contribute to desirable outcomes for Medicare beneficiaries with pain (15,16,26,64,65). For example, in a systematic review with meta-analysis, yoga had beneficial effects in patients with low back pain as well as in patients with osteoarthritis, kyphosis, rheumatoid arthritis, and fibromyalgia (53). Additionally, in other systematic reviews with meta-analyses, Tai chi was found to be effective for chronic pain associated with osteoarthritis and other chronic pain syndromes (54,55). It is worth mentioning that Medicare Advantage plans are considered to have higher payment thresholds, provide coverage in more settings and for treatments not included in fee-for-service Traditional Medicare such as yoga classes and Tai chi (7,66).

### Implications for Future Research

Given the growing proportion of Medicare beneficiaries who are enrolled in Medicare Advantage and that Medicare Advantage enrollees are predominantly racial and ethnic minorities, it is imperative to investigate differences in coverage policies for nonpharmacological pain treatments in Traditional Medicare versus Medicare Advantage and whether any differential coverage exacerbates well-documented racial inequities in pain management and treatment (67,68). Our findings that research on nonpharmacological pain therapy use is limited among Asian Americans, Pacific Islanders, American Indians, or Alaska Natives and that racial and ethnic differences were commonly assessed between non-Hispanic Whites and Black/African Americans highlight the need for studies that have more racial and ethnic diversity and which offer more detailed reporting of data on minority populations. Furthermore, given that much of the literature on pain treatment disparities has mainly focused on opioid prescribing, it is imperative to expand the scope of research on racial equity in pain treatment to include nonopioid and nonpharmacological approaches (69–73).

There is a role for qualitative inquiry to investigate patient and provider knowledge, beliefs, and preferences of nonpharmacological pain treatments so that tailored education and other interventions can be designed and deployed. A recent study conducted by Garrett et al. (2021) also highlights the importance of understanding patient beliefs and preferences regarding nonpharmacological pain management approaches through qualitative research, which can provide valuable insights for designing tailored interventions (74). For instance, surveys or interviews could be conducted to explore the factors that influence patient and provider decisions to use acupuncture or other nonpharmacological pain therapies. Research is also needed to evaluate the efficacy and cost-effectiveness of guideline-recommended pain therapies in later life, which might inform policymakers to broaden Medicare coverage for nonpharmacological pain treatments through reimbursement for new therapies such as those offered in Medicare Advantage and not Traditional Medicare (eg, Tai chi). Furthermore, interdisciplinary pain programs and group-based mindfulness are increasingly tested in RCTs and appear to confer some benefit (5). Randomized controlled trials or observational studies could compare the outcomes and costs of different nonpharmacological pain treatments among older adults with chronic pain. Extending coverage to more types of pain beyond primarily low back pain and relaxing utilization management restrictions such as visit limits also deserve consideration. For example, current Medicare coverage limits a patient’s receipt of PT and chiropractic care annually. This is particularly limiting for patients with chronic pain who may experience multiple exacerbations of pain throughout a calendar year and may not be able to receive services based on coverage restrictions. Opportunities also exist to investigate the uptake of acupuncture following the new CMS policy to provide reimbursement for acupuncture services. Lastly, the studies included in the review included data from 1990 to 2018, indicating a gap in the literature on the current trends of nonpharmacological treatment modalities and how these trends were affected during the acute phase of the coronavirus pandemic and beyond.

### Strengths and Limitations

This scoping review is strengthened by the rigorous methods that were followed in conducting the study. For example, we searched an expansive list of databases and various sources of gray literature to identify studies to be considered for review. Additionally, we evaluated a comprehensive set of guideline-recommended nonpharmacological pain treatments including those for which Medicare coverage is currently unavailable and would be unobservable in studies that rely on claims data alone. However, this review has some limitations. First, because our goal was to characterize the evidence on nonpharmacological pain treatment use within the Medicare program, studies that focused on older adults with health insurance other than Medicare (e.g., VHA) were excluded. As such, these findings may not generalize outside the Medicare population. Second, we excluded RCTs and CMS demonstration projects, which may have provided meaningful information about practice and policy levers that may influence the use and outcomes of nonpharmacological pain treatment in Medicare. Furthermore, the efficacy, safety, and cost-effectiveness of these treatment modalities among Medicare beneficiaries were beyond the scope of this study. Lastly, as this was a scoping review rather than a systematic review, no evaluation of the quality of individual studies was undertaken. Although the present scoping review is an important first step in summarizing the state of the existing literature, systematic reviews that build upon the current findings are an appropriate next step.

## Conclusion

In summary, we undertook this review to identify and summarize the scope of studies that directly address nonpharmacological pain treatment primarily among older adults in the U.S. The combination of the high prevalence of chronic pain conditions in later life, rapid growth in Medicare enrollment, and worsening opioid crisis, which is increasingly affecting older adults (75), heightens the importance of documenting the existing evidence regarding the characteristics and context of nonpharmacological pain management in Medicare. This scoping review, which included publications from the past 3 decades, identified the need for future research that uses more recent data and addresses contemporary issues such as the expansion of Medicare Advantage, equitable access to evidence-based pain management, promulgation of practice guidelines for managing pain, and evaluation of the impacts of new Medicare coverage for nonpharmacological approaches to pain treatment. Developing this understanding will promote the advancement of clinical practice and policymaking toward equitable and comprehensive pain management for older adults.

## Supplementary Material

igad085_suppl_Supplementary_Table
