# Supplementary material for: Treatment Patterns and Population Characteristics of Nonpharmacological Management of Chronic Pain in the United States’ Medicare Population: A Scoping Review
Source: Innov Aging. 2023 Aug 11;7(10):igad085. doi: 10.1093/geroni/igad085 (PMC10714895; doi:10.1093/geroni/igad085)
Supplement: igad085_suppl_Supplementary_Table [file igad085_suppl_supplementary_table.docx]

**Supplementary Table 1**: Summary of treatment modalities examined in studies included in this review

| **First Author, Year** | **Pharmacologic treatment modalities** | | | **Noninvasive nonpharmacologic treatment identified from pain treatment guidelines and recommendations** | | | | | | | | | | | | | | **Additional noninvasive nonpharmacologic treatment modalities** | | | | | | | | | | | | | | **Surgical treatment**  **Modalities** |
| --- | --- | --- | --- | --- | --- | --- | --- | --- | --- | --- | --- | --- | --- | --- | --- | --- | --- | --- | --- | --- | --- | --- | --- | --- | --- | --- | --- | --- | --- | --- | --- | --- |
|  | Opioid medications | Non-opioid pain medications | Other medications | Exercise | Massage | Tai chi | Yoga | Mindfulness-based stress reduction | Multidisciplinary rehabilitation | Relaxation techniques | Acupuncture | Laser therapy | Biofeedback | Psychological therapies | Manipulative therapy | Electrical stimulation | Myofascial release | Diet and nutrition | Self-care strategies | Occupational therapy | Ice and heat therapy | Therapeutic Ultrasound | Home remedies | Chinese medicine | chelation therapy | Homeopathy | Ayurvedic medicine | Naturopathy | Speech Therapy | Prayers/spiritual healing | Stones/jewelry |  |
| Albert, 2008a |  |  |  | X |  |  |  |  |  | X |  |  |  |  |  |  |  | X | X |  | X |  | X |  |  |  |  |  |  |  |  |  |
| Albert, 2008b | X | X | X | X | X |  |  | X |  | X |  |  |  |  | X |  |  | X |  |  | X |  | X |  |  |  |  |  |  |  |  |  |
| Astin, 2000 |  |  |  |  | X |  |  |  |  |  | X |  |  |  | X |  |  | X |  |  |  | X | X | X | X | X | X | X |  |  |  |  |
| Davis, 2015 |  |  |  |  |  |  |  |  |  |  |  |  |  |  | X |  |  |  |  |  |  |  |  |  |  |  |  |  |  |  |  |  |
| Davis, 2019 |  |  |  |  |  |  |  |  |  |  |  |  |  |  | X |  |  |  |  |  |  |  |  |  |  |  |  |  |  |  |  |  |
| Davis, 2021 | X |  |  |  |  |  |  |  |  |  |  |  |  |  | X |  |  |  |  |  |  |  |  |  |  |  |  |  |  |  |  | X |
| De Heer, 2016 |  |  |  |  |  |  |  |  |  |  |  |  |  |  | X |  |  |  |  |  |  |  |  |  |  |  |  |  |  |  |  |  |
| Fritz, 2011 |  |  |  |  |  |  |  |  |  |  |  |  |  |  | X |  |  |  |  |  |  |  |  |  |  |  |  |  |  |  |  |  |
| Gell, 2017 |  |  |  |  |  |  |  |  |  |  |  |  |  |  | X |  |  |  |  | X |  |  |  |  |  |  |  |  | X |  |  |  |
| Hufstader, 2009 | X | X |  |  | X |  |  |  |  |  | X |  |  |  | X |  |  | X |  |  |  |  | X |  |  |  |  |  |  | X |  |  |
| Jordan, 2000 | X | X | X | X | X |  |  | X |  | X |  |  | X |  | X |  |  | X |  |  | X |  |  |  |  |  |  |  |  |  | X |  |
| Karmali, 2020 | X |  |  |  |  |  |  |  |  |  |  |  |  | X | X |  |  |  |  | X |  |  |  |  |  |  |  |  |  |  |  |  |
| Latham, 20080 |  |  |  |  |  |  |  |  |  |  |  |  |  |  | X |  |  |  |  | X |  |  |  |  |  |  |  |  |  |  |  |  |
| Lieberz, 2020 | X |  | X |  |  |  |  |  |  |  |  |  |  |  | X |  |  |  |  |  |  |  |  |  |  |  |  |  |  |  |  |  |
| Ly, 2020 | X | X | X |  |  |  |  |  |  |  |  |  |  |  | X |  |  |  |  |  |  |  |  |  |  |  |  |  |  |  |  |  |
| Mayer-Oakes, 1992 |  |  |  |  |  |  |  |  |  |  |  |  |  |  | X |  |  |  |  | X |  |  |  |  |  |  |  |  |  |  |  |  |
| Musich, 2020 | X |  | X | X |  |  |  |  |  |  |  |  |  |  | X |  |  |  |  |  |  |  |  |  |  |  |  |  |  |  |  |  |
| Ngo, 2009 |  |  |  |  |  |  |  |  |  |  |  |  |  |  | X |  |  |  |  | X |  |  |  |  |  |  |  |  |  |  |  |  |
| Sclafani, 2017 |  |  | X |  |  |  |  |  |  |  |  |  |  |  | X |  |  |  |  |  |  |  |  |  |  |  |  |  |  |  |  | X |
| Standaert, 2020 |  |  | X |  |  |  |  |  |  |  |  |  |  |  | X |  |  |  |  |  |  |  |  |  |  |  |  |  |  |  |  | X |
| Stevans, 2017 | X | X | X | X |  |  |  |  |  |  |  |  |  |  | X |  |  | X |  |  |  |  |  |  |  |  |  |  |  |  |  | X |
| Thorpe, 2021 |  |  |  |  |  |  |  |  |  |  |  |  |  |  | X |  |  |  |  | X |  |  |  |  |  |  |  |  |  |  |  |  |
| Weeks, 2016 | X | X | X |  |  |  |  |  |  |  |  |  |  |  | X |  |  |  |  |  |  |  |  |  |  |  |  |  |  |  |  | X |
| Weigel, 2010 |  |  |  |  |  |  |  |  |  |  |  |  |  |  | X |  |  |  |  |  |  |  |  |  |  |  |  |  |  |  |  |  |
| Weigel, 2012 |  |  |  |  |  |  |  |  |  |  |  |  |  |  | X |  |  |  |  |  |  |  |  |  |  |  |  |  |  |  |  |  |
| Weigel, 2013 |  |  |  |  |  |  |  |  |  |  |  |  |  |  | X |  |  |  |  |  |  |  |  |  |  |  |  |  |  |  |  |  |
| Weigel, 2014a |  |  |  |  |  |  |  |  |  |  |  |  |  |  | X |  |  |  |  |  |  |  |  |  |  |  |  |  |  |  |  |  |
| Weigel, 2014b |  |  |  |  |  |  |  |  |  |  |  |  |  |  | X |  |  |  |  |  |  |  |  |  |  |  |  |  |  |  |  |  |
| Weiner, 2006 |  |  | X |  |  |  |  |  |  |  |  |  |  |  | X |  |  |  |  |  |  |  |  |  |  |  |  |  |  |  |  | X |
| Whedon, 2012 |  |  |  |  |  |  |  |  |  |  |  |  |  |  | X |  |  |  |  |  |  |  |  |  |  |  |  |  |  |  |  |  |
| Whedon, 2013 |  |  |  |  |  |  |  |  |  |  |  |  |  |  | X |  |  |  |  |  |  |  |  |  |  |  |  |  |  |  |  |  |
| Whedon, 2021a |  |  |  |  |  |  |  |  |  |  |  |  |  |  | X |  |  |  |  |  |  |  |  |  |  |  |  |  |  |  |  |  |
| Whedon, 2021b | X |  |  |  |  |  |  |  |  |  |  |  |  |  | X |  |  |  |  |  |  |  |  |  |  |  |  |  |  |  |  |  |
| Whedon, 2022 | X |  |  |  |  |  |  |  |  |  |  |  |  |  | X |  |  |  |  |  |  |  |  |  |  |  |  |  |  |  |  |  |
| **Total** | **12** | **6** | **10** | **5** | **4** | **0** | **0** | **2** | **0** | **3** | **2** | **0** | **1** | **1** | **33** | **0** | **0** | **6** | **1** | **6** | **3** | **1** | **4** | **1** | **1** | **1** | **1** | **1** | **1** | **1** | **1** | **6** |
| *Notes:* X= Use of treatment modality (*The color is added for visual display and does not provide unique information*); Manipulation Therapy= Physical therapy, Chiropractic care and Spinal Manipulation therapy; non-opioid pain medications= NSAIDs (Non-steroidal anti-inflammatory drugs), acetaminophen etc.; Other medications= Muscle relaxants, antidepressant, anticonvulsants, knee, or spinal injections etc. | | | | | | | | | | | | | | | | | | | | | | | | | | | | | | | | |
